# Corrigendum

**DOI:** 10.1002/ams2.643

**Published:** 2021-04-08

In Ito et al.,^1^ the following errors were published.

In Figure 1, the legends for Case 1 and Case 2 were incorrect. The blue line should have been Case 1, and the orange line, Case 2. The corrected version of Figure 1 is below:

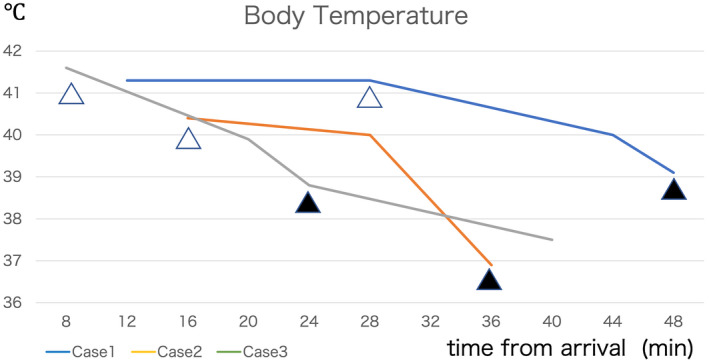

Figure 2 was incorrectly published without a black strip covering the subject’s eyes. The corrected version of Figure 2 is below:

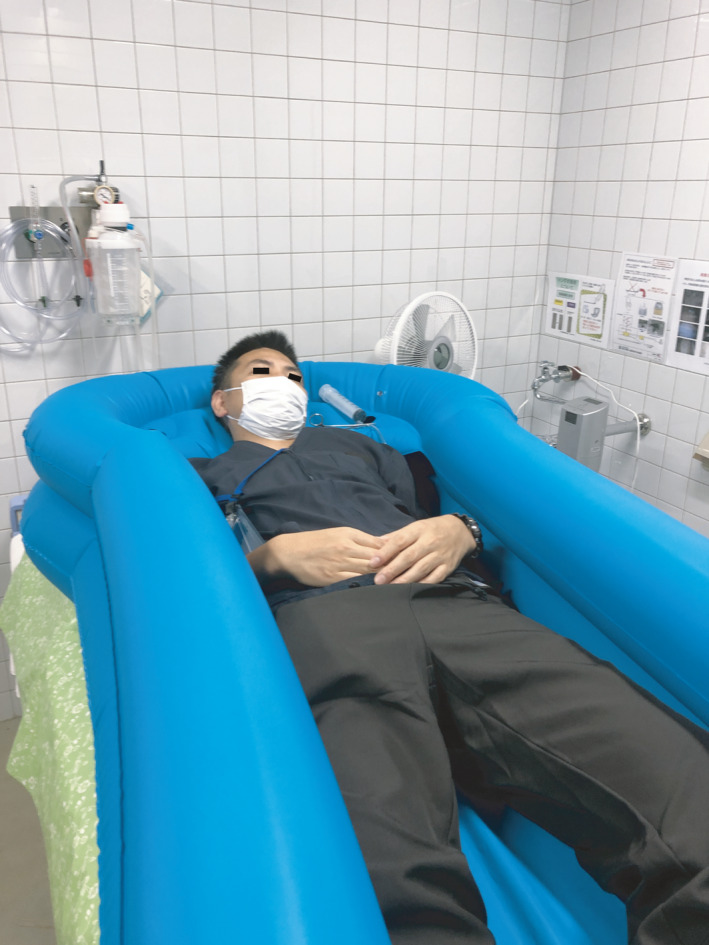

The online version of this article was corrected.

The authors apologize for these errors and any confusion they may have caused.
